# Supplementary material for: HIV-1 Vpu induces neurotoxicity by promoting Caspase 3-dependent cleavage of TDP-43
Source: EMBO Rep. 2024 Sep 6;25(10):18. doi: 10.1038/s44319-024-00238-y (PMC11467202; doi:10.1038/s44319-024-00238-y)
Supplement: Supplementary file 2 — Appendix [file 44319_2024_238_MOESM2_ESM.pdf]

APPENDIX

HIV-1 Vpu induces neurotoxicity by promoting Caspase 3-dependent cleavage of TDP-43

Jiaxin Yang *et al.*

*Corresponding author. Email: wwei6@jlu.edu.cn

Table of Contents

Appendix Figure S1 ········ Page 2
Appendix Figure S2 ········ Page 3
Appendix Figure S3 ········ Page 4
Appendix Figure S4 ········ Page 5
Appendix Figure S5 ········ Page 6
Appendix Figure S6 ········ Page 7
Appendix Table S1 ········ Page 8

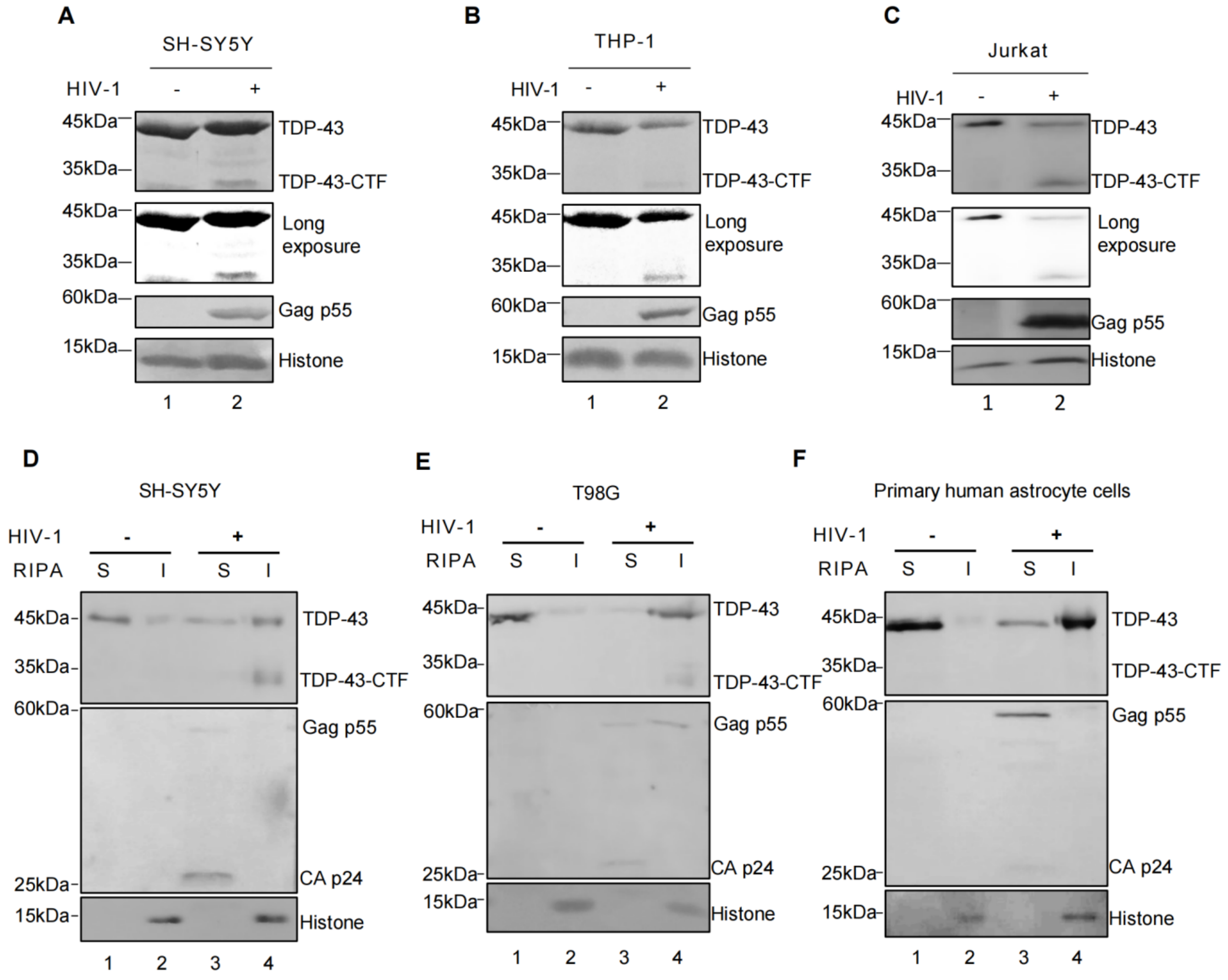

Appendix Figure S1. Infection with HIV-1 induces the cleavage and aggregation of TDP-43 in different cell lines. (A-C) Lysates of SH-SY5Y, THP-1 and Jurkat cells were assessed by Western blotting after infection with the HIV-1-ΔEnv-EGFP-VSV-G virus using the indicated antibodies. **(D-F)** Cell fractionation analysis of nerve cells infected with the HIV-1-ΔEnv-EGFP-VSV-G virus.

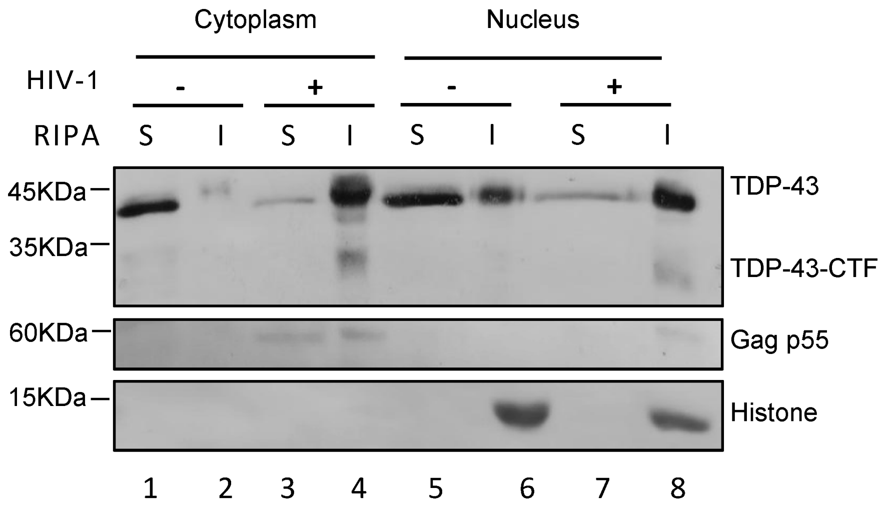

Appendix Figure S2. The solubility of TDP-43 proteins in the nuclear and cytoplasmic compartments of cells before and after HIV-1 infection. HIV-1-infected cells or control cells were subjected to subcellular fractionation to isolate cytosolic and nuclear components, followed by the use of a RIPA/urea fractionation assay for separation of the RIPA soluble fraction (S) and the RIPA insoluble fraction (I). The obtained samples were analysed by Western blotting using the indicated antibodies.

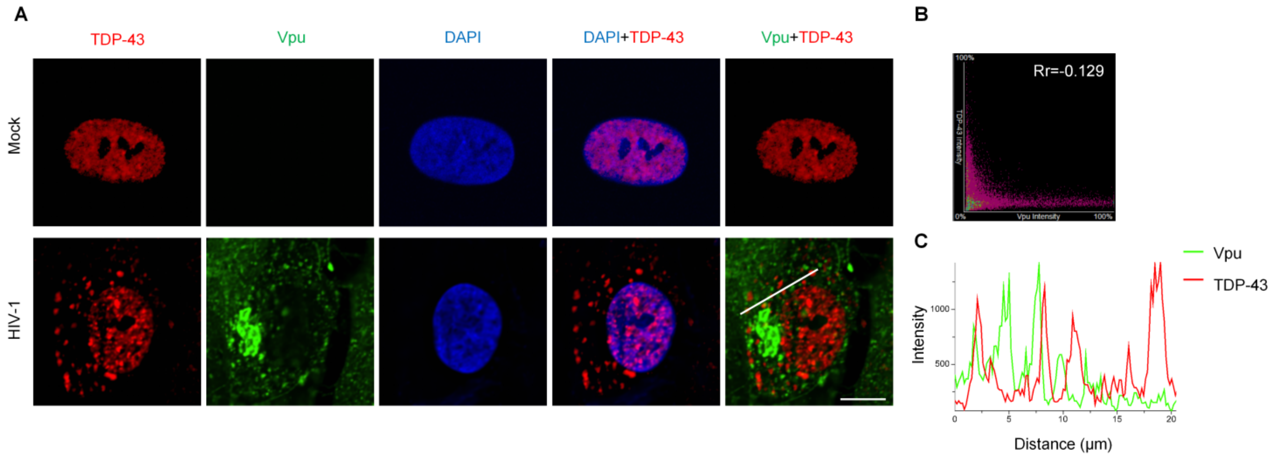

Appendix Figure S3. Colocalization analysis of Vpu and TDP-43. (A) Immunofluorescence images of primary human astrocyte cells infected with HIV-1. Antibodies against TDP-43 and Vpu were used. Scale bar, 10 μm. **(B)** Colocalization scatter plot image of Vpu and TDP-43. Rr, Pearson's colocalization coefficient. **(C)** Plots of intensity along the white line from the image in (A).

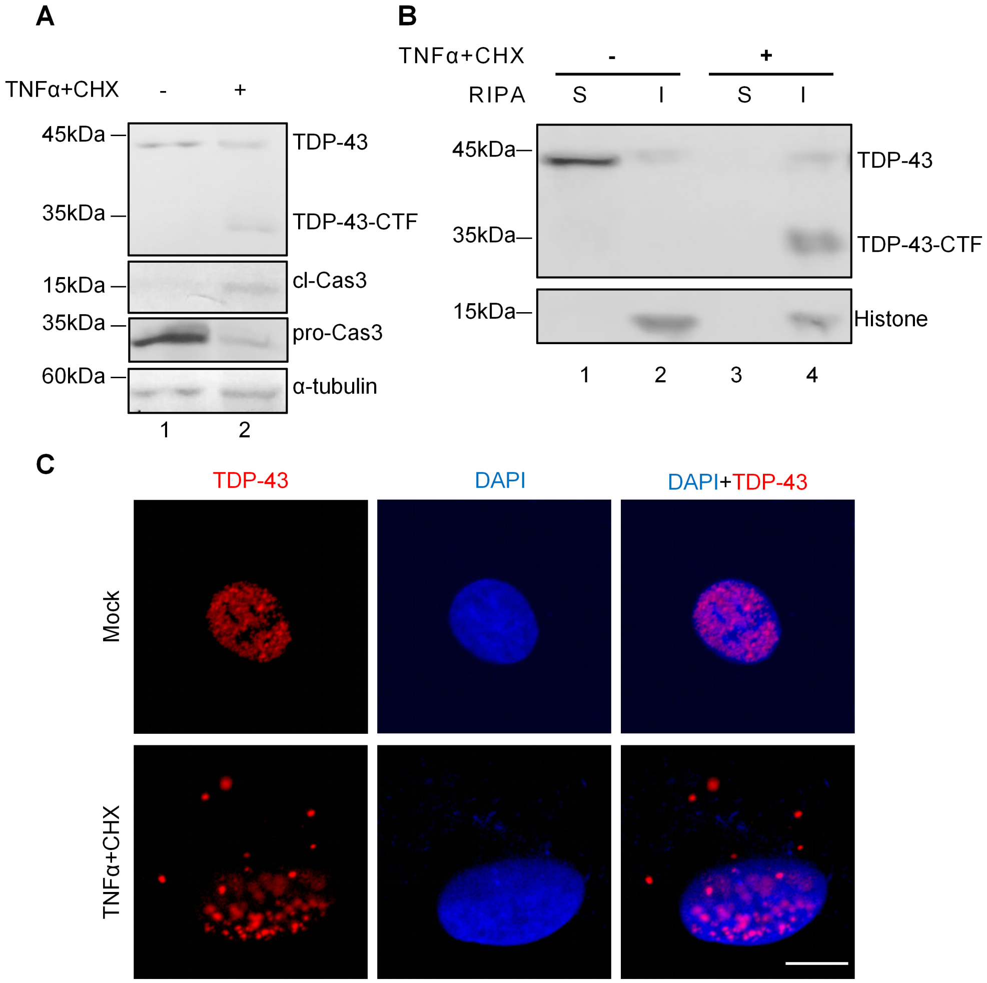

Appendix Figure S4. TNFα/CHX treatment induces the cleavage and aggregation of TDP-43. **(A)** Primary human astrocytes were treated with TNFα (20 ng/ml) and CHX (10 μg/ml) for 10 hours. The cell lysates were assessed by Western blotting using the indicated antibodies. CHX, cycloheximide. **(B)** Cell fractionation analysis of T98G cells treated with TNFα and CHX. **(C)** Immunofluorescence images of primary human astrocyte cells treated with TNFα and CHX.

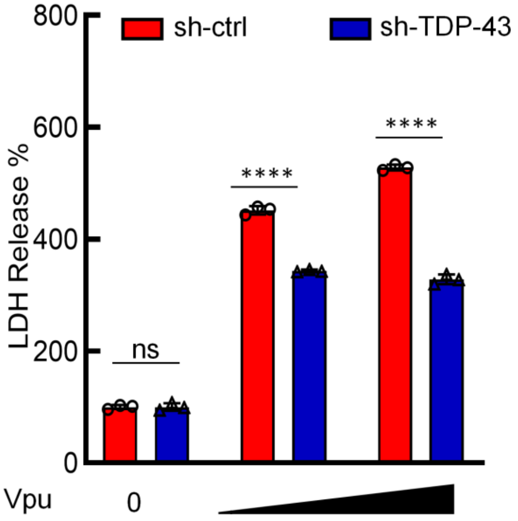

Appendix Figure S5. Downregulating TDP-43 relieves Vpu cytotoxicity. VR1012-Vpu-Myc (0, 0.2 or 0.4 μg) was transfected into control or TDP-43-knockdown cells. 72 hours posttransfection, the supernatants were collected to measure the level of LDH release using an LDH assay kit. Data information: data are presented as mean ± SEM. ANOVA, n=3 biological replicates. ns, not significant,****P < 0.0001.

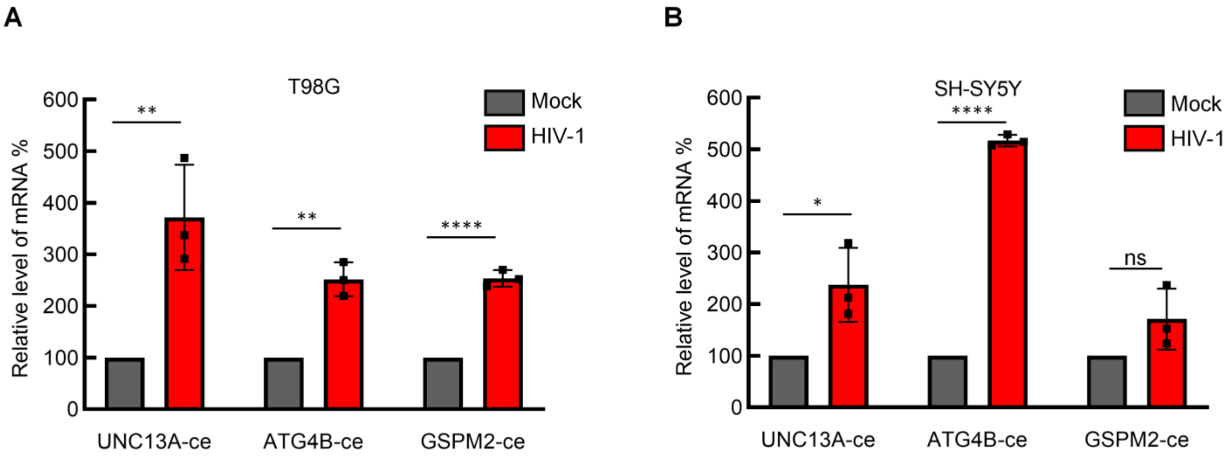

Appendix Figure S6. HIV-1 infection induces the accumulation of cryptic exons in nerve cells. Total RNA was extracted at 72 hours post HIV-1 infection. After reverse transcription,real-time quantitative PCR were performed to detect the mRNA level of cryptic exons. **(A)****UNC13A-ce P=0.01, **ATG4B-ce P=0.0014, ****GSPM2-ce P<0.0001. **(B)***P=0.0292, ****P<0.0001, ns,not significant. Data information: data are presented as mean ± SEM. ANOVA, n=3 biological replicates.

Appendix Table S1

qPCR primers	
UNC13A-ce forward primer	TGGATGGAGAGATGGAACCT
UNC13A-ce reverse primer	GGGCTGTCTCATCGTAGTAAAC
ATG4B-ce forward primer	TGTGTCTGGATGTGAGCGTG
ATG4B-ce reverse primer	TCTAGGGACAGGTTCAGGACG
GSPM2-ce forward primer	AGTGGACATGTGGTGGTAAGAA
GSPM2-ce reverse primer	GCTTCAAAGAATGACACGCCA
