# Supplementary material for: HIV-1 Vpu induces neurotoxicity by promoting Caspase 3-dependent cleavage of TDP-43
Source: EMBO Rep. 2024 Sep 6;25(10):18. doi: 10.1038/s44319-024-00238-y (PMC11467202; doi:10.1038/s44319-024-00238-y)
Supplement: Supplementary file 9 — Expanded View Figures [file 44319_2024_238_MOESM9_ESM.pdf]

Expanded View Figures

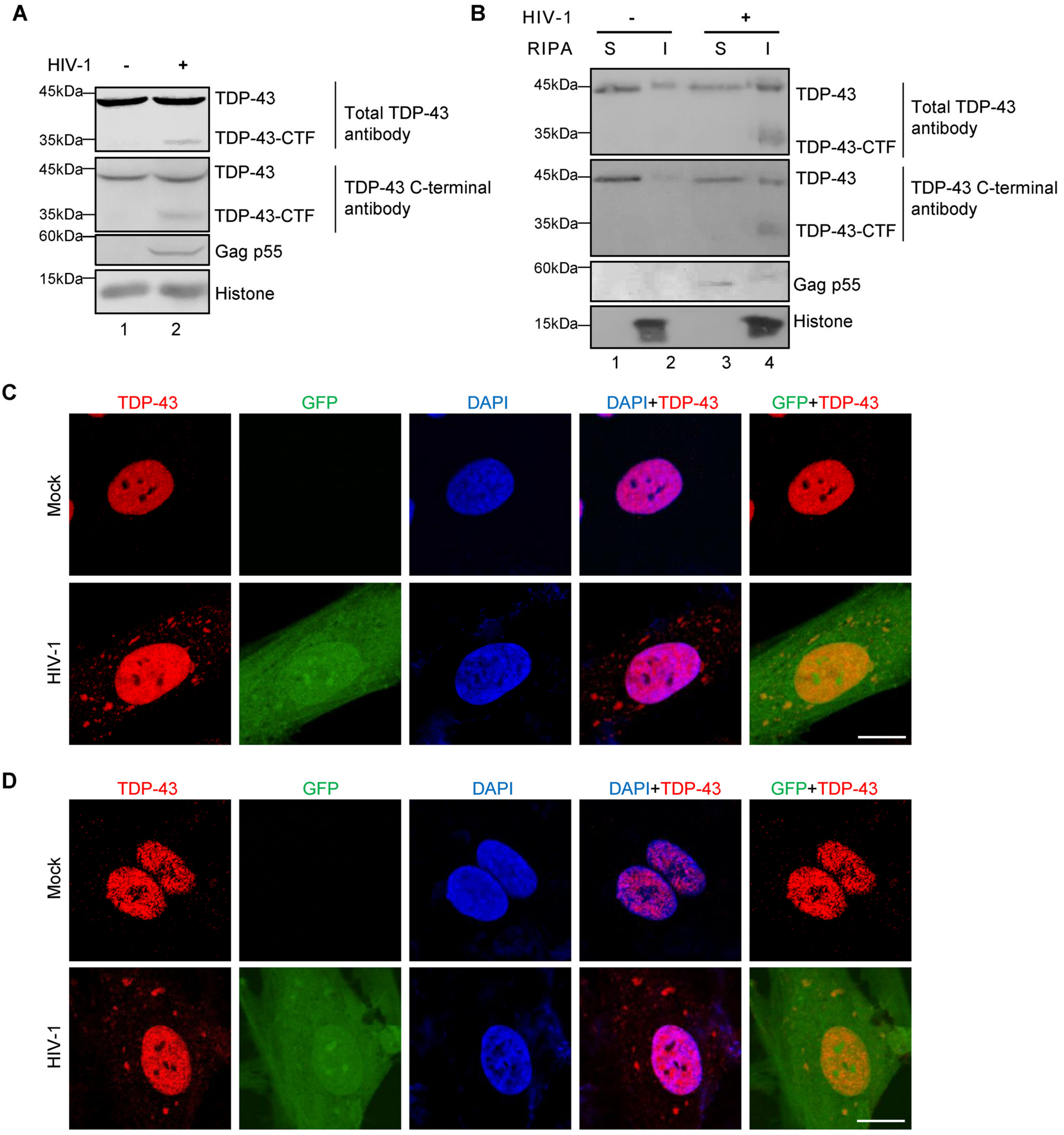

Figure EV1. HIV-1 induces the cleavage and cytoplasmic aggregation of TDP-43.

(**A**) Western blotting of primary human astrocyte cells infected with the HIV-1-ΔEnv-EGFP-VSV-G virus. TDP-43 antibodies were used to detect endogenous TDP-43. CTF, C-terminal fragment. (**B**) Cell fractionation analysis of SH-SY5Y cells infected with the HIV-1-ΔEnv-EGFP-VSV-G virus. S soluble, I insoluble. (**C**, **D**) Immunofluorescence images of primary human astrocyte cells infected with the HIV-1-ΔEnv-EGFP-VSV-G virus. Total TDP-43 antibody (Proteintech, 10782-2-AP) (**C**) and TDP-43 (C-terminal) antibody (Proteintech, 12892-1-AP) (**D**) were used to detect TDP-43. Scale bar, 10 µm.

© The Author(s)

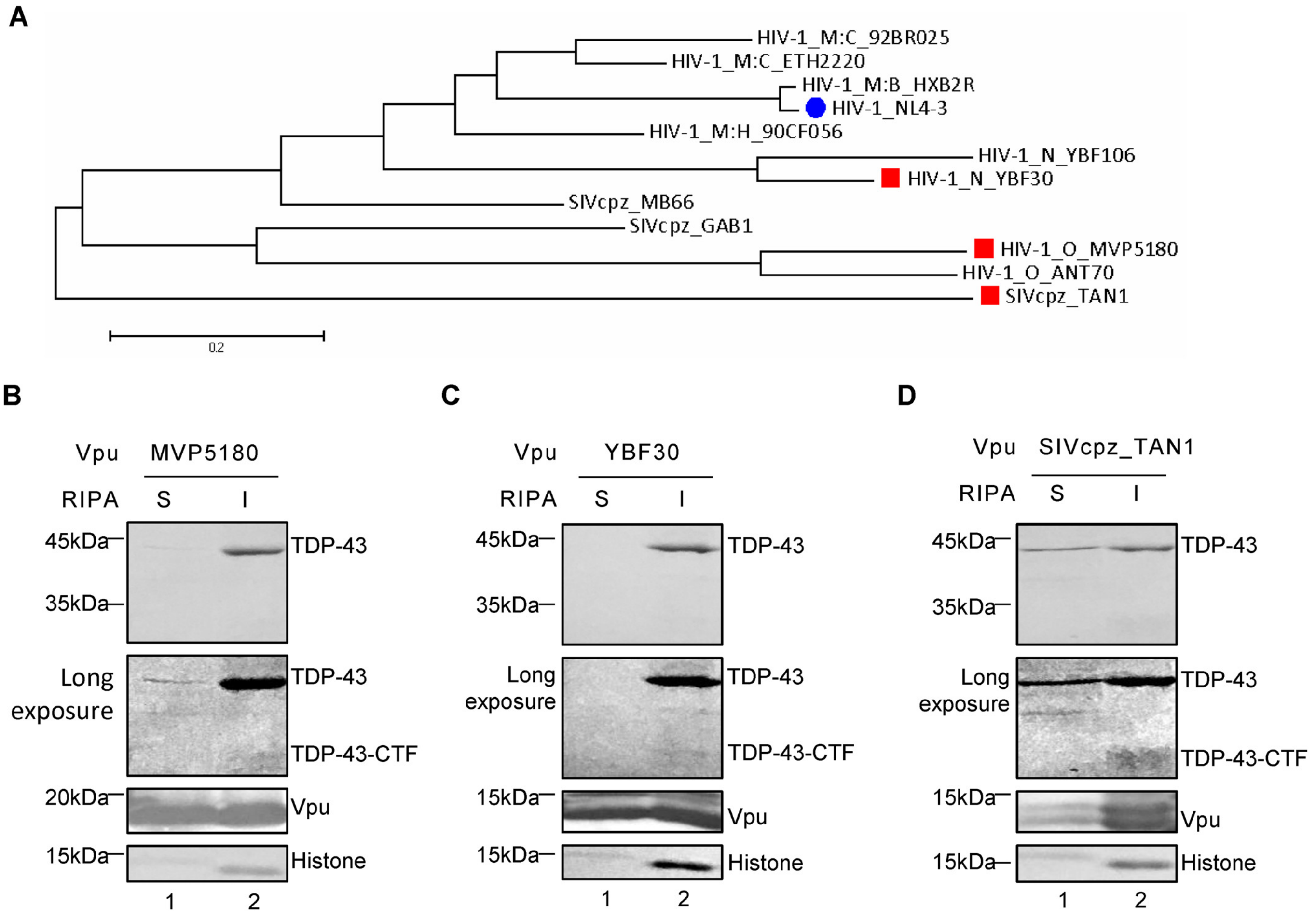

Figure EV2. The aggregation of TDP-43 is induced by Vpu derived from different strains of HIV-1 and SIVcpz_TAN1.

(**A**) The amino acid sequences of the indicated HIV-1/SIV Vpu proteins were retrieved from the HIV database (www.hiv.lanl.gov). Evolutionary history was inferred using the neighbor-joining method, and evolutionary analyses were conducted in MEGA7. The tree is drawn to scale, with branch lengths and evolutionary distances used to infer the phylogenetic tree shown in the same units. (**B–D**) Cell fractionation analysis of HEK293T cells transfected with expression plasmids as shown. S soluble, I insoluble. The asterisk indicates the cleavage product of TDP-43.

 © The Author(s)

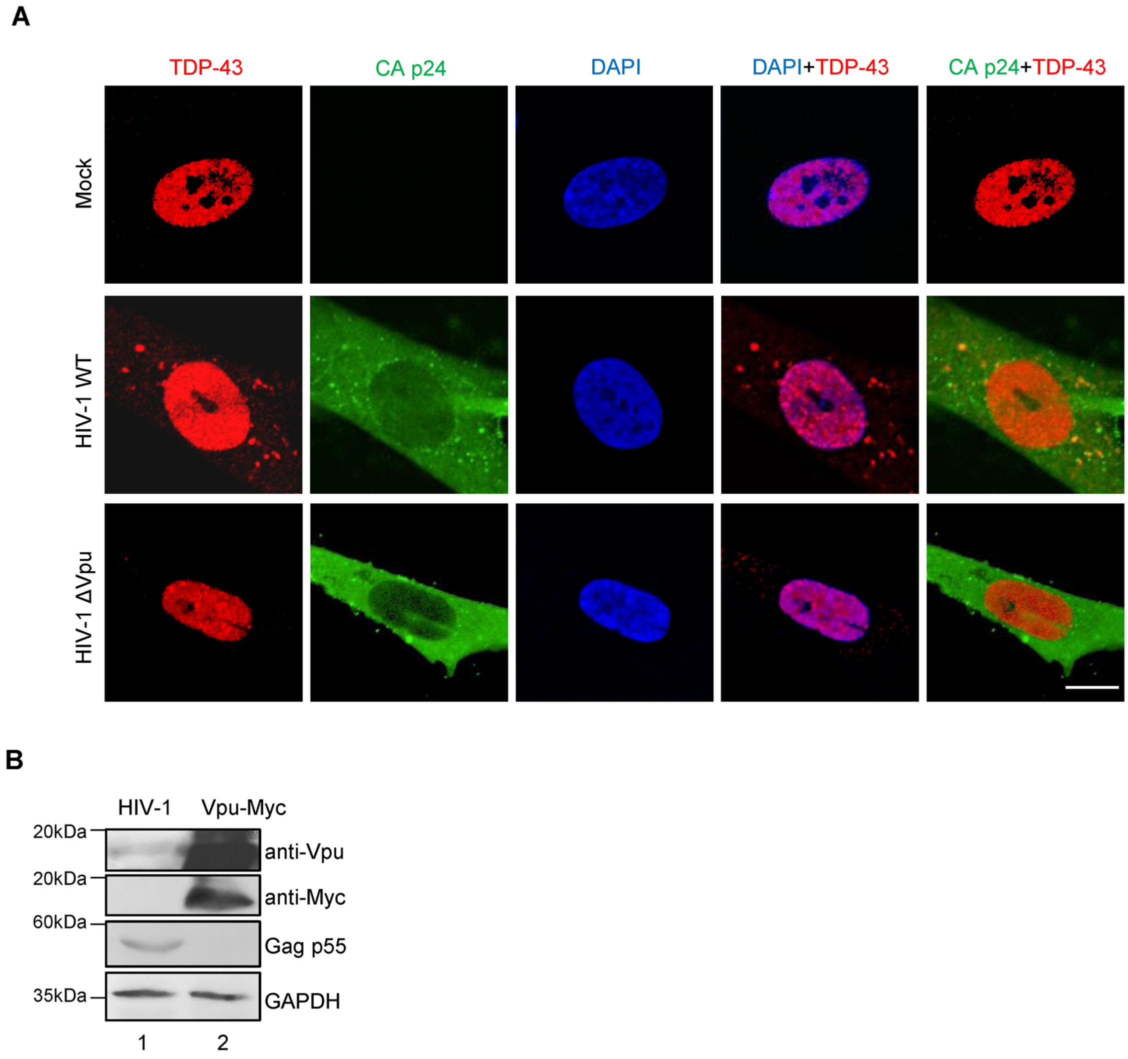

Figure EV3. Vpu is essential for the cytoplasmic translocation of TDP-43 induced by HIV-1.

(**A**) Immunofluorescence images of primary human astrocyte cells infected with the HIV-1-ΔEnv-VSV-G virus. CA p24 antibodies were used to detect HIV-1. Scale bar, 10 μm. (**B**) Western blotting of HEK293T cells infected with the HIV-1-ΔEnv-EGFP-VSV-G virus or transfected with VR1012-Vpu-Myc. CA p24 antibody was used to detect HIV-1 infection. A Myc-tag antibody was used to detect Vpu overexpression. A Vpu antibody was used to detect Vpu protein in all the samples.

© The Author(s)

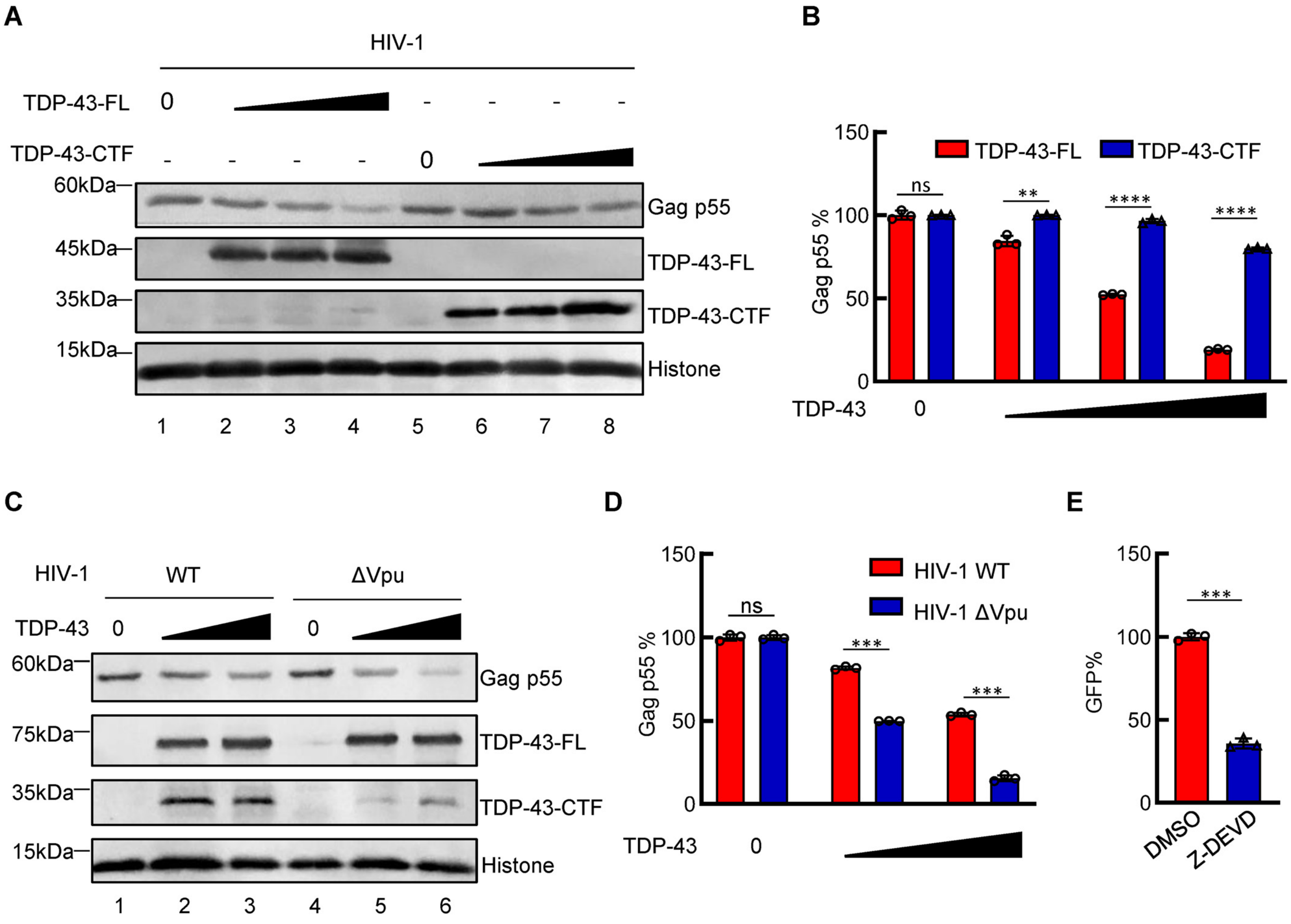

Figure EV4. The cleavage of TDP-43 is facilitated by Vpu to augment HIV-1 transcription.

(**A**, **B**) Western blotting of HEK293T cells cotransfected with HIV-1-ΔEnv-EGFP and different doses of the VR1012-TDP-43-FL-HA- or VR1012-TDP-43-CTF-HA expression vectors (0, 0.25, 0.5, or 1 μg). A CA p24 antibody was used to detect Gag p55 in cell lysates. HA-tag antibodies were used to detect TDP-43-FL and TDP-43-CTF. The bar graph shows the relative percentage of Gag p55 according to grey analysis, ns not significant, $^{**}P = 0.0012$, $^{****}P < 0.0001$. (**C**, **D**) HEK293T cells were cotransfected with wild-type or ΔVpu HIV-1-ΔEnv plasmids and a low level of pmC1-TDP-43 (0, 0.2 or 0.4 μg). The cells were collected for Western blotting with the indicated antibodies. The relative levels of Gag p55 according to grey analysis are shown in the bar graph. ImageJ was used for grey analysis, ns not significant, 0.2 μg group $^{***}P = 0.0003$, 0.4 μg group $^{***}P = 0.0018$. (**E**) The infection efficiency of the HIV-1-ΔEnv-EGFP-VSV-G virus was detected by flow cytometry. The HIV-1-ΔEnv-EGFP-VSV-G virus was packaged in HEK293T cells, and then the cells were treated with DMSO or Z-DEVD-FMK (20 μM) posttransfection. The supernatants were collected for infection, and infectivity was measured 48 h after viral infection using flow cytometry to detect GFP-positive cells, $^{***}P = 0.0007$. Data information: data are presented as mean ± SEM. ANOVA, $n = 3$ biological replicates.

 © The Author(s)

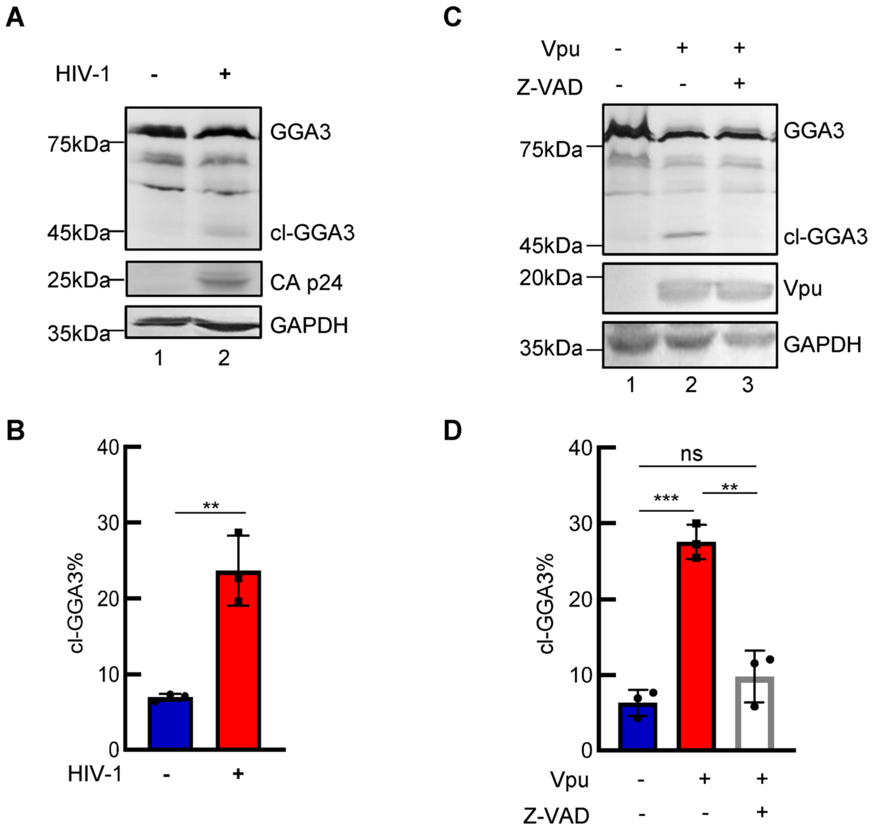

Figure EV5. HIV-1 Vpu induces cleavage of GGA3 in a Caspase-dependent manner.

(**A**) Western blotting of HEK293T cells transfected with the HIV-1-ΔEnv-EGFP plasmid to detect endogenous GGA3 using a GGA3 antibody. (**B**) The bar graph shows the percentages of the relative band intensities for cl-GGA3 relative to total GGA3 according to grey analysis. $^{**}P = 0.0034$. (**C**) Western blotting of HEK293T cells transfected with VR1012-Vpu-Myc and treated with DMSO or 20 μM Z-VAD-FMK at 4 h posttransfection. cl, cleaved. (**D**) The bar graph shows the percentages of the relative band intensities for cl-GGA3 relative to total GGA3 according to grey analysis. $^{***}P = 0.0002$, $^{**}P = 0.0017$, ns not significant. Data information: data are presented as mean ± SEM. ANOVA, $n = 3$ biological replicates.
